# Tuberculous Lymphadenitis Mimicking Nodal Metastasis in Follicular Variant Papillary Thyroid Carcinoma

**DOI:** 10.1155/2016/5623104

**Published:** 2016-09-26

**Authors:** Marc Gregory Yu, Jenny Maureen Atun

**Affiliations:** ^1^Section of Endocrinology, Diabetes and Metabolism, Department of Medicine, Philippine General Hospital, Manila, Philippines; ^2^Department of Pathology, Philippine General Hospital, Manila, Philippines

## Abstract

Tuberculous (TB) lymphadenitis can mimic cervical node metastasis from papillary thyroid carcinoma (PTC) since the distribution and appearance of affected lymph nodes are similar. We present the case of an asymptomatic 50-year-old Filipino who sought consult for a gradually enlarging anterior neck mass and a single palpable cervical lymph node. Preoperative workup suggested a thyroid malignancy with nodal metastasis. He underwent total thyroidectomy with node dissection where histopathology confirmed follicular variant- (FV-) PTC. Lymph node examination, however, revealed TB lymphadenitis, and the patient was given standard antimycobacterial therapy. This is the first documented case in Southeast Asia, a high TB burden region. This is also the first report involving FV-PTC, which has features between those of conventional PTC and follicular thyroid carcinoma. The case suggests that, in endemic areas, TB should be a differential in the etiology of cervical lymphadenopathy in PTC patients. In developed countries, this differential diagnosis is also valuable because of the increasing incidence of HIV and TB coinfection. Proper preoperative evaluation is important and needs to be highlighted in the formulation of local guidelines.

## 1. Introduction

Papillary thyroid carcinoma (PTC) is sometimes associated with cervical lymphadenopathy on presentation [1, 2]. Tuberculous (TB) lymphadenitis, the most common form of extrapulmonary tuberculosis, is usually indistinguishable from lymph node metastasis due to PTC since the distribution and appearance of affected lymph nodes tends to be similar [3]. In a region with high TB burden such as the Philippines, a proper preoperative evaluation is needed to distinguish these two entities and provide appropriate treatment.

## 2. Case Presentation

A 50-year-old Filipino male presented at the outpatient clinic with a seven-year history of a gradually enlarging anterior neck mass. Prior to this consult, he was apparently healthy with good functional capacity. On physical examination, a firm, nontender mass measuring 5 × 4 × 2 cm was palpated at the left thyroid lobe without any bruits. An associated 1 × 1 × 0.5 cm rubbery, nontender lymph node was also palpated at the left cervical area. The rest of the examination was otherwise unremarkable. The patient denied any hypo- or hyperthyroid symptoms or compressive symptoms such as dyspnea, dysphagia, or odynophagia. Likewise, he denied symptoms of chronic cough, night sweats, weight loss, or fever. There was no personal history of prior treatment or known exposure to TB, no previous head and neck irradiation, and no family history of thyroid disease.

Laboratory testing revealed a normal hemogram and serum biochemistry. Thyroid function was normal with free thyroxine (fT4) levels at 21.3 pmol/L (NV 12–24 pmol/L) and thyroid stimulating hormone (TSH) levels at 0.59 *μ*IU/mL (NV 0.3–3.8 *μ*IU/mL). A thyroid ultrasound (US) showed a 6.5 × 5 × 3 cm solid mass at the left lobe with a singular 1.2 × 1 × 0.5 cm cervical lymph node.

**Figure 1:**
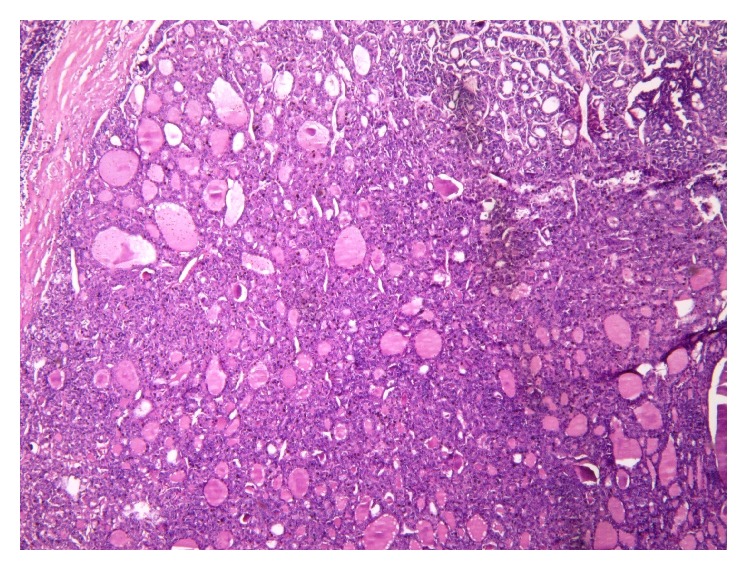
Scanning view of the thyroid mass showing tumor cells forming varied-sized follicles with densely eosinophilic colloid within the follicle lumen, consistent with follicular variant papillary thyroid carcinoma (magnification: 40x).

Preoperative fine-needle aspiration biopsy (FNAB) was performed for the thyroid mass revealing PTC. The patient underwent total thyroidectomy with neck dissection, with an unremarkable perioperative course. Histopathological examination confirmed follicular variant- (FV-) PTC, measuring 6.5 cm in widest dimension at the left lobe with extension to the isthmus. There was no extension beyond surgical margins or into extrathyroidal tissue (Figure 1).

**Figure 2:**
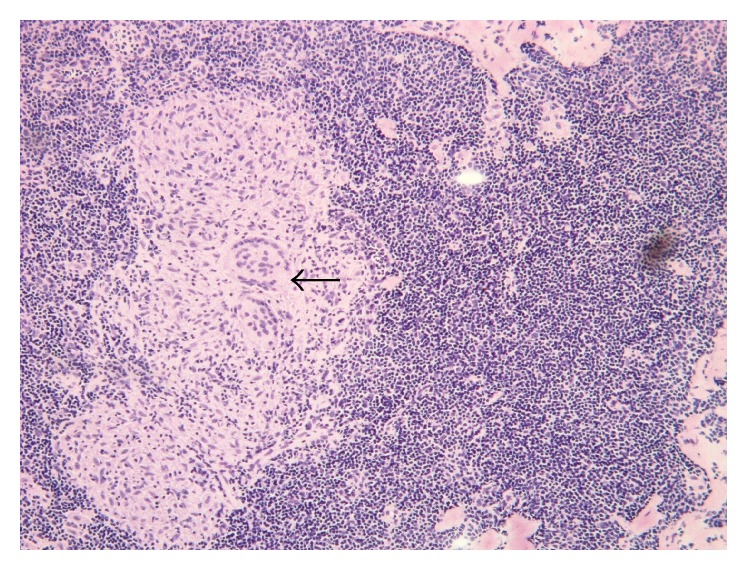
Low power view of the lymph node showing a granuloma composed of epithelioid histiocytes with abundant eosinophilic cytoplasm and multinucleated Langhans giant cell formation (black arrow), consistent with a tuberculous etiology (magnification: 400x).

Histopathology of the lymph node, however, yielded findings of chronic granulomatous inflammation with caseation necrosis and Langhans type giant cells consistent with a tuberculous etiology (Figure 2). A chest X-ray plus two consecutive acid-fast (AFB) stained sputum samples were negative for concomitant pulmonary TB. The patient was started on a four-drug intensive phase regimen of isoniazid, rifampicin, pyrazinamide, and ethambutol for two months, followed by a two-drug maintenance phase regimen of isoniazid and rifampicin for the next four months.

The patient was followed up at the outpatient clinic six weeks after surgery. The surgical site had satisfactorily healed and antimycobacterial therapy was well-tolerated. He underwent adjuvant high-dose (100 mci) radioactive iodine treatment with no untoward complications and is currently on levothyroxine suppression therapy.

## 3. Discussion

PTC is the most common type of thyroid malignancy, constituting over 80% of all thyroid cancer cases [4]. It preferentially metastasizes to regional lymph nodes, with cervical lymphadenopathy described in 23–56% of PTC cases at initial presentation [1, 2]. Neck dissection is the current standard of care in PTC patients with clinically positive neck nodes. Although the procedure is relatively reliable and safe, it can still lead to substantial postoperative complications and cosmetic problems. A careful preoperative evaluation is therefore paramount so that patients need not undergo an unwarranted neck dissection for other conditions [5].

The coexistence of PTC and TB lymphadenitis has been described in several case reports from India, Korea, Japan, and the US [3, 6–8]. In a case series of PTC patients with lymphadenopathy, 72% (18/25) were eventually diagnosed with TB lymphadenitis after an initial consideration of metastasis from PTC [9]. In another study of 1693 patients with PTC and lymphadenopathy, 28 had TB lymphadenitis. Of these, seven also had concomitant nodal metastasis from PTC [10].

Our patient initially presented as a straightforward case of PTC manifesting as an asymptomatic gradually enlarging anterior neck mass with an associated solitary cervical lymph node. Due to the documented malignancy plus the proximity of the lymph node, our primary consideration for the lymphadenopathy was metastatic spread. Although US is a reasonably sensitive diagnostic method, it is difficult to truly ascertain the etiology of lymphadenopathy as the distribution and sonographic appearance are virtually identical in TB adenitis and cervical node metastasis. Both preferentially occur in the supraclavicular area or posterior triangle of the neck [3], appearing as round and hypoechoic structures with frequent intranodal cystic necrosis and calcifications [11]. In addition, current guidelines do not recommend routine preoperative FNAB of lymph nodes in PTC, which is staged clinically and radiologically. Thus, without a high index of suspicion, the diagnosis of TB lymphadenitis can be easily missed.

In a surveillance report by the WHO-Western Pacific Region, the Philippines ranks ninth among the highest TB-burdened countries in the world. TB adenitis is the most common manifestation of extrapulmonary TB, which has an estimated prevalence of 1.1% in the country [12]. A histopathologic examination remains the most accurate test for diagnosis [13], but preoperative FNAB can also be done with a sensitivity of 46–90% [14]. Additionally, polymerase chain reaction (PCR) of the aspirate can be employed to increase the sensitivity and specificity [15]. In retrospect, a prompt TB workup in our patient could have identified the disease preoperatively, leading to earlier initiation of antimycobacterial therapy and sparing the patient an unnecessary neck dissection.

Considering the high TB prevalence in Southeast Asia, this is the first documented case of its kind in the region. Moreover, in contrast to other reports dealing with either conventional type PTC (C-PTC) or mixed conventional and macrofollicular PTC, this is also the first case involving pure FV-PTC, the most common PTC subtype. Our case is significant in the sense that FV-PTC has a unique clinical behavior, representing an intermediate entity with features that are between those of C-PTC and follicular thyroid carcinoma (FTC). As compared to C-PTC, FV-PTC has been shown to have a lower rate of lymph node metastasis and a higher rate of distant metastasis [16].

## 4. Conclusion

The case illustrates that cervical lymphadenopathy in a patient with PTC may not always indicate metastatic spread from the disease. In developing countries, TB should be considered an important differential in the etiology of cervical lymphadenopathy in a patient with PTC. Likewise, in developed countries, this differential diagnosis is also valuable because of the increasing incidence of HIV and TB coinfection [17]. Proper preoperative evaluation remains the cornerstone of providing appropriate treatment. In endemic countries, guidelines may need to be revised accordingly.
